# Artificial intelligence in the pediatric echocardiography laboratory: Automation, physiology, and outcomes

**DOI:** 10.3389/fradi.2022.881777

**Published:** 2022-09-09

**Authors:** Minh B. Nguyen, Olivier Villemain, Mark K. Friedberg, Lasse Lovstakken, Craig G. Rusin, Luc Mertens

**Affiliations:** ^1^Division of Cardiology, Department of Paediatrics, The Hospital for Sick Children, University of Toronto, Toronto, ON, Canada; ^2^Department of Pediatric Cardiology, Baylor College of Medicine, Houston, TX, United States; ^3^Centre for Innovative Ultrasound Solutions and Department of Circulation and Medical Imaging, Norwegian University of Science and Technology, Trondheim, Norway

**Keywords:** pediatric cardiology, pediatric echocardiography, echocardiography, pediatrics, artificial intelligence

## Abstract

Artificial intelligence (AI) is frequently used in non-medical fields to assist with automation and decision-making. The potential for AI in pediatric cardiology, especially in the echocardiography laboratory, is very high. There are multiple tasks AI is designed to do that could improve the quality, interpretation, and clinical application of echocardiographic data at the level of the sonographer, echocardiographer, and clinician. In this state-of-the-art review, we highlight the pertinent literature on machine learning in echocardiography and discuss its applications in the pediatric echocardiography lab with a focus on automation of the pediatric echocardiogram and the use of echo data to better understand physiology and outcomes in pediatric cardiology. We also discuss next steps in utilizing AI in pediatric echocardiography.

## Introduction

Artificial intelligence (AI), the study and development of computational algorithms that mimic human cognitive functions such as learning and thinking, is used in non-medical fields to assist with automation and decision-making (1). There are multiple tasks AI is designed for that could improve the quality, interpretation, and clinical application of echocardiographic data at the level of the sonographer, echocardiographer, and clinician. Machine learning (ML), algorithms whose goal is to learn patterns from data to improve at a given task, is a field within AI that has been applied to many tasks in medical research. ML algorithms often require some engineering of the input features (i.e., the process of creating new variables from the data such as extracting pixel density, peak velocity from a spectrogram, or a measurement from an image) prior to developing the model which can be both time consuming and challenging especially for large high dimensional datasets like images and videos. Deep learning (DL) is a subset of ML algorithms that allow more flexibility in approximating the underlying structure of the data which leads to less feature engineering requirements to obtain accurate predictions. While this approach is especially appealing for high dimensional data such as echocardiograms, this flexibility is at the cost of increased complexity which has its own shortcomings (e.g., ‘black-box' decision-making and computational cost) (2).

Advances in pediatric cardiac imaging have proven challenging due to the complexity of pediatric heart disease and the impact of growth. The potential for ML in pediatric cardiology, especially in the pediatric echocardiography laboratory, is very high. While there are several excellent recent reviews that have highlighted the clinical applications of AI in medicine and cardiology, reviews specific to AI applied in the pediatric echocardiography laboratory are lacking (1, 3–5). In this state-of-the-art review, we identify needs unique to the pediatric echocardiography laboratory that could be addressed by AI, the recent applications of ML in pediatric echocardiography, and perspectives on future directions of AI in the pediatric echocardiography laboratory.

## Part I: Optimizing the pediatric echocardiogram

Echocardiography is one of the fundamental technologies that helps guide the diagnosis and treatment of children with congenital heart disease (CHD). AI, especially DL, has been implemented in other fields where it has excelled in tasks using unstructured data (e.g., raw images and video clips) such as facial recognition and automated driving (2). Here, we describe the recent literature on using deep learning to automate and optimize echocardiographic acquisition, image optimization, measurements, and diagnosis in pediatric cardiology (Tables 1, 2).

**Table 1:** Key AI literature in pediatric echocardiography.

**Study**	**Type of ML**	**Description**
**Acquisition/optimization**		
Komatsu et al. (6)	CNN	Detection and labeling of cardiac structures in a fetal US
Diller et al. (7)	GAN	Denoising and artifact removal in congenital heart disease echocardiograms
**View classification**		
Arnaout et al. (8)	CNN and FCN	Classify fetal echocardiographic views
Gearhart et al. (9)	CNN	Classify transthoracic echocardiographic views
**Segmentation**		
Guo et al. (10)	FCN	Segment and measure left atrial/ventricular structures
**Measurements**		
He et al. (11)	CNN	Segment Left Ventricle in different views and estimate LV EF
**Diagnosis**		
Chotzoglou et al. (12)	GAN	Screen fetal echocardiograms for abnormal cardiac structures
Arnaout et al. (8)	CNN and FCN	Screen fetal echocardiograms for congenital heart disease
Wang et al. (13)	CNN	Diagnose septal defects on pediatric transthoracic echocardiograms
**Cardiac phenotypes**		
Meza et al. (14)	Hierarchical Clustering	Identify parameters that distinguish LV obstructive disease
Garcia-Canadilla et al. (15)	MKL and K-means Clustering	Identify high-risk phenotypes for adverse events in dilated cardiomyopathy

CNN, convolutional neural network; US, ultrasound; GAN, generative adversarial network; FCN, fully convolutional network; LV, left ventricle; EF, ejection fraction; MKL, multiple kernel learning.

**Table 2:** Selected machine learning approaches to pediatric echocardiography.

**Algorithm**	**Description**
**Deep learning**	
Convolutional neural network	A network architecture that is especially strong in detecting underlying patterns in imaging data for both unsupervised and supervised tasks. Though quite versatile, often requires large datasets to be reliable
Fully convolutional network	A type of CNN that is especially useful for segmentation (e.g., tracing) tasks, but can be computationally inefficient. Example: U-Net
Generative adversarial network	Unique architecture that is employed in unsupervised and certain supervised tasks. Consists of two networks: a generator that creates synthetic data and a discriminator that tries to distinguish synthetic data from real data
**Conventional** **machine learning**	
Ensemble methods	Algorithms that employ several decision trees and averages their outputs to create a composite decision. Requires structured data (i.e., features are manually created) and can be computationally intense. Examples: Random Forest, XGBoost
Cluster analysis	A group of unsupervised learning techniques that create homogenous groups of observations based on similarities between features. Examples: Hierarchical, K-means
Multiple kernel learning	A kernel-based group of algorithms used in both supervised and unsupervised tasks including for non-linear dimensionality reduction (to determine the set of features that retain the most variability in the dataset)
Dimensionality reduction	Seeks to determine the set of features that retain the most variability in the dataset to find a good representation of the data with the least amount of variables. The resulting output is abstract and at best estimates the feature values

### Facilitating image acquisition and automating optimization

**Figure 1:**
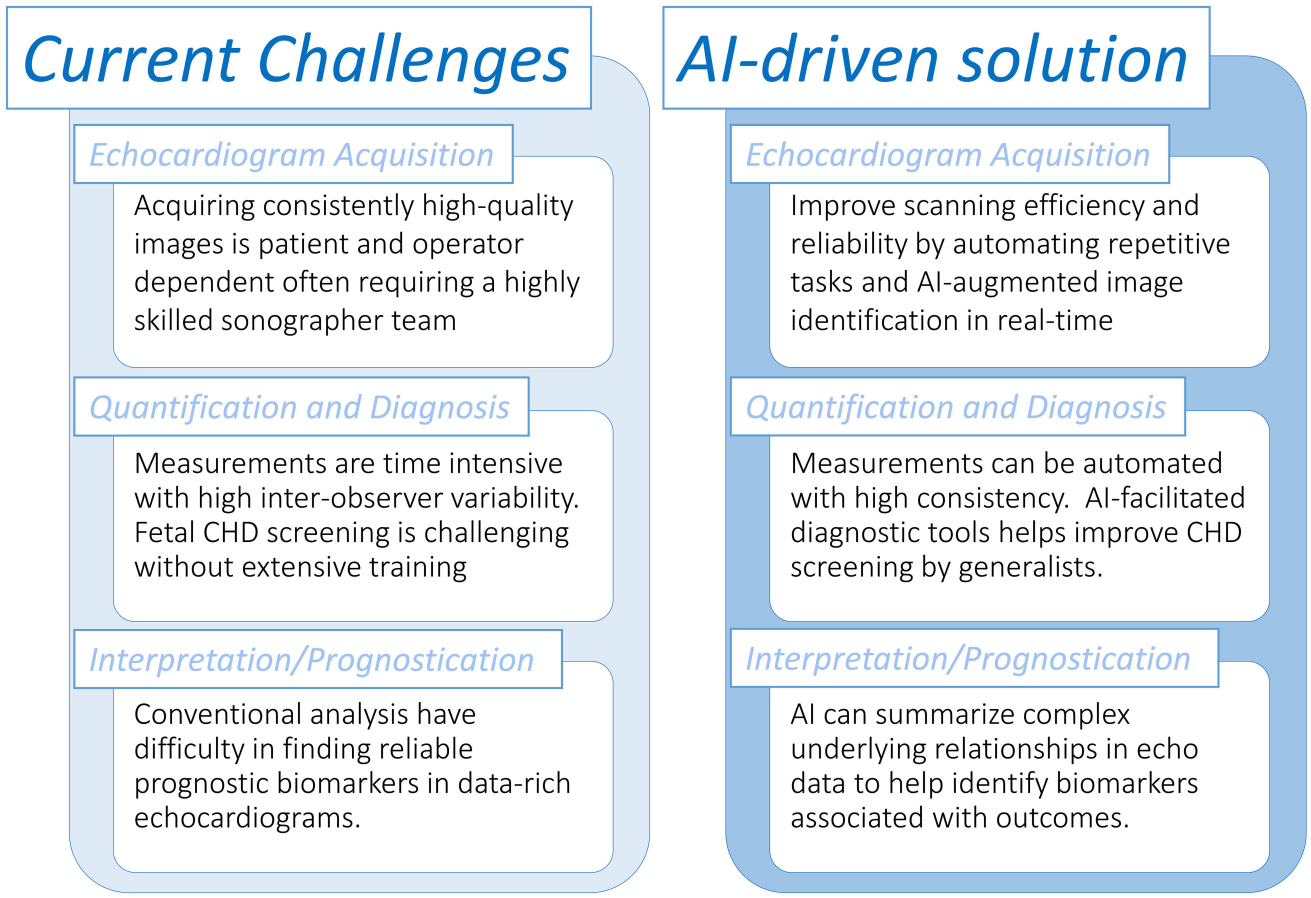
Overview of tasks in the pediatric echocardiography laboratory that could be assisted with artificial intelligence workflows.

In congenital and pediatric cardiology, the quality of ultrasound image acquisitions is guided by the proper implementation of published practice guidelines (16). However, despite best efforts to adhere to consensus standards, misdiagnoses may still occur as echocardiography is a highly operator-dependent technique. This may be related to errors at the acquisition level such as sonographic planes being incorrectly obtained or the defect visualized on screen but not recognized by the operator (17) (Figure 1). AI has been shown to achieve human-level performance in some medical imaging analysis tasks (18). This raises the potential for automating aspects of the pediatric ultrasound scan, including automated image identification and measurements in real-time. In fetal CHD screening, proof of concept studies using AI tools during the acquisition phase have been shown to positively impact the efficiency and quality of the fetal examination compared to a standard manual scan. Recently, Matthew et al. showed that using AI-embedded tools to reduce repetitive tasks (e.g., measuring fetal biometry and manually acquiring video clips of standard fetal views) may allow more attention to be directed to obtaining accurate morphological diagnoses (19). They used an ensemble of convolutional neural networks (CNN), a type of DL algorithm that is versatile in performing tasks related to images and videos, that were trained for anatomic measurements and image classification (20). Komatsu et al. (6) also found that CNNs could be trained to achieve an automatic detection of each cardiac substructure in fetal ultrasound videos, and showed this could be applied to assist in detecting cardiac structural abnormalities.

Transthoracic echocardiographic measurements and interpretation are heavily reliant on optimal probe positioning and insonation angle (16). Østvik et al. (21) trained a CNN to automatically identify if the operator has positioned the probe correctly to obtain optimal angles for seven different cardiac views (e.g., parasternal long axis, apical 2/3/4 chamber). They demonstrated the CNN's ability to do this in real time such that it could facilitate optimal acquisition at the bedside. Issues of image optimization for automation can be more challenging in fetal and pediatric echocardiography, despite having generally better image quality. Indeed, contrary to adult cardiology, multiple ultrasound probe types can be used at different frequencies and different frame rates. This issue of acquisition heterogeneity is especially troublesome in AI as the impact on image quality and the ensuing lack of homogeneity is detrimental in AI-model training if not accounted for (22). For this purpose, AI-assisted feedback systems have been proposed to facilitate optimization of parameters including depth, gain and frequency (23). Additionally, AI-based denoising and artifact removal tools have been recently developed for transthoracic echocardiographic imaging in congenital heart disease that can further standardize image quality (7). This allows a new generation of standardized high-quality images that can be used for AI-based tools for measurement, segmentation, and classification.

While these proof-of-concept studies support that AI can address a strong clinical need within echocardiography, each study has developed their own proprietary means to integrate their AI algorithms and operator interface with the ultrasound scanner. Extensive collaboration and development with industry is needed before these novel innovations can be incorporated into readily available commercial packages.

### Automating echocardiography laboratory tasks

After image acquisition and prior to the comprehensive interpretation of an echocardiogram, several intermediate steps need to be manually performed including view classification, segmentation of cardiac structures (e.g., “tracing” a clinically relevant structure within an image), and other quantitative measurements that rely on view classification and segmentation [e.g., ejection fraction (EF)].

While identifying an echocardiographic image to an experienced operator is a simple task, it is because training a sonographer or cardiologist in finding the correct view is often the first step in understanding echocardiography. Subsequently, training an algorithm to classify echocardiographic views is an important first step in creating an AI workflow, especially for automation tasks (2). This has been demonstrated to be feasible in adult studies (21, 24, 25). More recently, Arnaout et al. used 1,326 fetal echocardiograms to train a CNN to identify five standard fetal views (e.g., three-vessel view, left ventricular outflow tract view) with an AUC range of 0.72–0.88 (8). Furthermore, Gearhart et al. used a similar approach to perform automatic image view classification on 12,067 individual transthoracic pediatric echocardiographic images (9). The authors showed this model identified 28 preselected views with 90% accuracy.

In echocardiography, we perform segmentation of cardiac structures to assess abnormalities in cardiac morphology (e.g., left ventricular end diastolic diameter to assess for dilation) and to be used in other quantitative measurements (e.g., end systolic/diastolic left ventricular area to derive ejection fraction). When an AI algorithm is tasked to use an image to segment a cardiac structure, it needs to be trained on a pre-labeled set of training images, supervised learning. The accurate labeling (i.e., establishing the ground truth) of this dataset, which is often manually performed, is a key aspect to creating a strong model. However, in clinical medicine, such labels are not always clear-cut. For example, if a researcher was interested in developing a model to facilitate the echocardiographic screening of pediatric hypertrophic cardiomyopathy (HCM), the current guidelines on the diagnosis of HCM recommend a ventricular wall thickness *z*-score of >2.5 as a potential cutoff to screen asymptomatic children (26). Yet, it is unclear which z-score criteria to use (e.g., Detroit, Boston, PHN) and there may be inconsistency in the measurement itself (e.g., determining how to exclude right ventricular muscle bundles for interventricular septal diameter) all of which lead to imperfect labeling and thus a less accurate model. With that said, segmentation tasks with clearly defined labels have been proven to be comparable to manual segmentation in adult echocardiographic studies (25). However, in pediatrics sample size and variability in cardiac morphology are common challenges to the development of pediatric-specific AI models. Guo et al. (10) was able to develop a pediatric-specific DL model that is based on a fully convolutional network (FCN), a type of CNN that is designed for segmentation tasks common in medical imaging, to segment and perform measurements on the left atrium and left ventricle. They developed novel methods to accommodate for variability in size and heart rate prevalent in children. Other methods are being developed to augment the abilities of deep learning on smaller datasets such as the use of generative adversarial networks (GAN) (27, 28). GANs generate simulated data based on the original training images to improve the model's ability to perform a certain task; Arafati et al. developed an FCN to perform segmentation of atrial and ventricular chambers in the 4-chamber view and used a GAN to augment the 450 adult echocardiograms used resulting in a dice metric of 86%−92%, a measure of the degree of overlap between the model's segmentation and the manual segmentation.

It is possible for a deep learning algorithm to replicate the steps a human would take to perform a quantification task. For example, Zhang et al. (25) developed a CNN that automatically identified the apical four chamber view, selected the frames that best represented end-diastole and end-systole, traced the LV endocardial border, and then derived an ejection fraction from those steps. In contrast, Ouyang et al. used a CNN architecture that considers both spatial and temporal features (i.e., spatiotemporal convolution) in predicting EF (Simpson's) and did not restrict the model predictions solely based on end-diastolic volumes derived by endocardial area segmentation. This permitted the model to have more freedom to derive its own spatiotemporal features leading to a more accurate and consistent prediction (29). The downside to this approach is that is less transparent than the former model whose pipeline makes each part of the segmentation process explicit. This model was recently adapted for children where it was retrained on a pediatric dataset to not only use apical four chamber views but also parasternal short views to estimate EF by 5/6 area length method with an *R*^2^ of 0.78 (11). This so-called ‘transfer learning' technique of fine-tuning a model previously trained on a different dataset to be optimized for a different task is a methodology unique to DL. In practice it allows the practitioner to develop a model with significantly less data, a problem that frequently occurs in pediatric cardiology (30, 31).

Apart from traditional methods to assess ventricular function, other functional measurements including strain imaging can be fully automated. However, compared to adult echocardiography labs the uptake of the method has been slower in pediatric cardiology (32). This can be explained by the anatomical variability present in congenital cardiology, the absence of dedicated post-processing software adjusting for different probe frequencies, and the limitations in frame rates relative to the higher heart rates present in younger children, especially in infants (33). Some of these limitations could be addressed using an AI-based approach to strain imaging. An adult study has recently demonstrated that, using B-mode image acquisitions, an AI algorithm could automatically derive global longitudinal strain measurements with minimal measurement variability (34). Applying AI-based automated strain measurements in children would potentially result in improving its applicability in pediatric heart disease and better understanding of the factors that influence strain imaging in children (35, 36).

Several of the studies in this section have obtained large multicenter retrospective datasets to train and test their algorithms. However, this does not preclude these algorithms from prospective real-world trials to test their efficacy and generalizability. Indeed, a number of these studies rely on open-source datasets which have its own set of limitations (e.g., variable quality and number of images, poor labeling, etc.) which could induce bias if not properly accounted for (22). To address this, imaging biobanks are being developed whose goal is to provide standardized medical image collections for the development of higher quality models (37). Furthermore, ML is the norm within the field of radiomics, the study of deriving and analyzing imaging biomarkers from conventional medical images to aid in clinical decision support systems, and there is a strong push toward the standardization of not only image acquisition but of the entire radiomics pipeline from the development of features and ML algorithms, to its implementation in the clinical workspace (e.g., the image biomarker standardization initiative) (38).

### Diagnosis of heart disease

**Figure 2:**
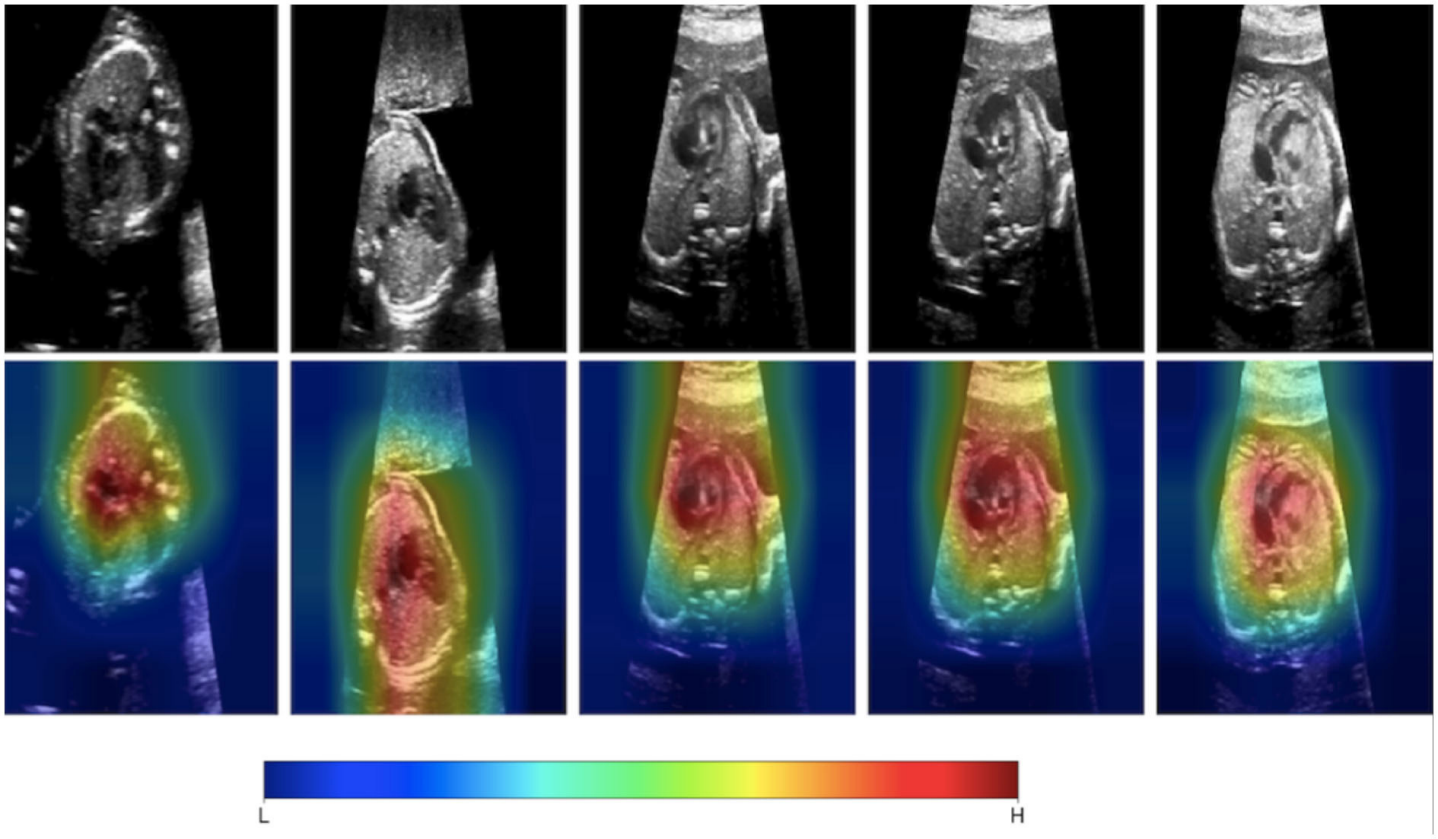
Gradient-weighted class activation mapping (Grad-CAM) helps us understand what aspects of an image are important in a deep learning model's decision-making. Chotzoglou et al. developed a DL model that classified fetal images as normal or abnormal cardiac anatomy. They used Grad-CAM mapping to demonstrate that the model identifies cardiac structures as important in understanding if the fetal ultrasound image represents a normal or abnormal heart. Figure adapted from Chotzoglou et al. (12).

The past 5 years have seen research efforts in developing AI-based tools to assist with congenital heart disease diagnosis. This would be useful as democratizing the diagnosis of significant congenital heart disease to non-expert echocardiography users would make the screening for CHD more efficient in particular in fetal echocardiography. First-line screening for CHD is typically provided by obstetricians during routine anatomic scans during the second trimester. Despite newer guidelines recommending a more comprehensive approach to screening including additional fetal views, CHD continues to be missed (17, 39). Having AI-based methods for screening would likely increase the detection rates for significant CHD. Chotzoglou et al. (12) were interested in developing an AI algorithm to screen for abnormal hearts on fetal echocardiography using a method called one-class anomaly detection. This method trains a deep learning model on normal echocardiographic data using generative adversarial networks and an autoencoder (α-GAN). Here, the GAN involved two separate networks: the first network (generator) creates simulated echocardiographic images based on the training data given to it, and a second model (discriminator) tries to discriminate if a given image is simulated or real. With this method, the α-GAN model was able to distinguish normal hearts from HLHS with an AUC of 0.81 when exposed to a fetal ultrasound dataset from a single center. To understand if the model was making clinically intelligible decisions, they applied a gradient-weighted class activation map to the model which visualizes which pixels the model deemed most important in detecting an abnormal image (Figure 2). This approach demonstrated that an AI-based model could help in screening for specific types of critical CHD. This will need to be tested for other types of CHD since HLHS was deliberately chosen as the initial lesion as it is grossly abnormal in the four-chamber view and thus identifiable from a single imaging plane. Arnaout et al. (8) further assessed the clinical applicability of deep learning as a screening tool by testing an ensemble of neural networks on a larger more heterogenous dataset of 107,823 images derived from multiple sources. They utilized a novel CNN algorithm for classification of fetal images and found an AUC of 0.95–0.99 discriminating normal from abnormal from a test set of many complex CHD.

As a decision support tool, AI could further help transthoracic echocardiography operators in diagnosing different types of CHD. For instance, two studies demonstrated the ability for DL models to diagnose septal defects including ASDs, VSDs, and AVSDs (13, 40). Additionally, Diller et al. (41) trained a CNN to classify apical 4-chamber and parasternal short-axis images as congenitally corrected transposition of the great arteries (ccTGA), d-TGA, and normal with an accuracy of 98%. Furthermore, they used transfer learning to adapt a CNN that was previously developed for biomedical image segmentation tasks to segment the endocardial border of the systemic ventricle (Dice score 0.79 for ccTGA). Of note, current error rates in CHD diagnosis in experienced pediatric echocardiography laboratories are extremely low relative to the number of studies performed, often with limited therapeutic impact. Instead, DL could prove more useful in developing models for specific interventions that diagnostic imaging is used to help with. For example, a CNN could be trained on echocardiographic data to accurately predict the correct atrial septal defect occlusion device, patent ductus arteriosus closure device, or pulmonary valve replacement device size and type. Using ML to assist in specific interventions in CHD is further elaborated below (Part II, subsection: *Congenital Heart Disease*).

## Part II: Machine learning to understand pediatric heart disease

While AI applications in echocardiography have largely focused on automation of different tasks, it can also be used to obtain a deeper statistical understanding of data by identifying cardiac phenotypes or associated factors to clinical outcomes. In the pediatric echocardiography lab, this could be used to understand how our non-invasive assessments reflect cardiac physiology.

### Explainability in machine learning

Supervised ML is where an algorithm's goal is to best predict the label (e.g., outcome) for a given set of pre-labeled observations. Some supervised algorithms, especially DL models, are often considered “black box” techniques in that it is difficult to understand how predictions are derived for a given model. The field of interpretable machine learning has been developed in order to explain model predictions which could help improve clinical acceptance of the ML model (42). For instance, for a given set of features (i.e., variables), we can compute how important an individual feature is by evaluating the impact on prediction accuracy in a dataset when those feature's values are not used in its prediction (e.g., permutation feature importance). To understand how features are used in a model, we can develop a global surrogate model to approximate the predictions of the original comprehensive model. Surrogate models provide a more transparent way of assessing feature usage (e.g., regression coefficient table in a logistic regression or feature value cutoffs in a decision tree) and by assessing the performance of the approximated model to the original comprehensive model, we can understand how accurate the surrogate model is (43, 44).

**Figure 3:**
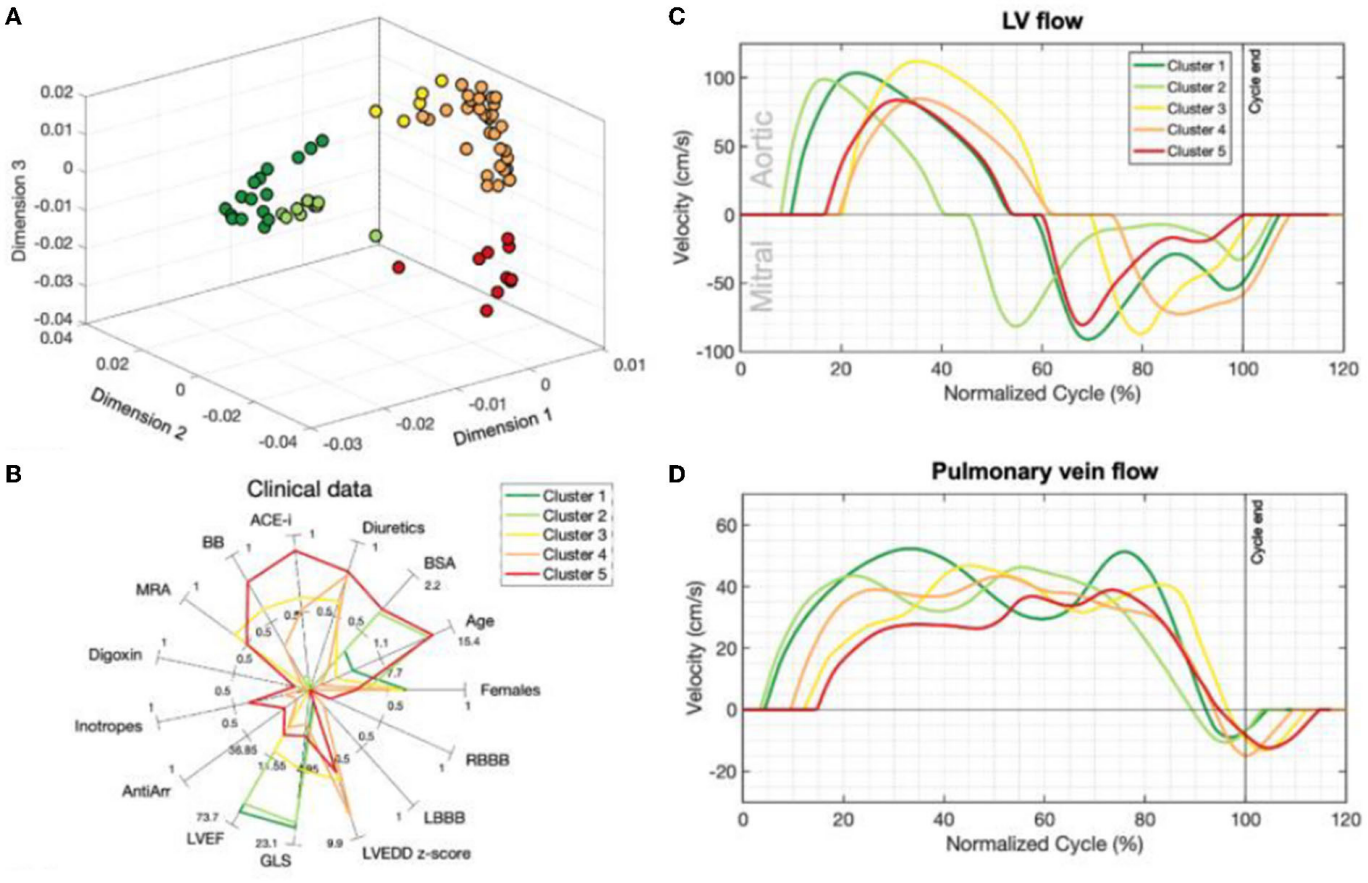
Unsupervised learning techniques such as dimensionality reduction and cluster analysis have improved our ability to identify imaging phenotypes [Garcia-Canadilla et al. (15)]. **(A)** Non-parametric dimensionality reduction techniques (multiple kernel learning) quantify the similarity between patients based on their echocardiographic inputs (Doppler velocity and ventricular strain tracings). This plot is a representation of how patients are positioned by their similarities based on dimensionality reduction. K-means clustering then identified five separate groups of patients based on these similarities (different colors represent different clusters). **(B)** Each cluster has clinically distinct characteristics. Clusters 1 and 2 were healthy volunteers. Clusters 3–5 were DCM patients. In particular, cluster 5 had the oldest patients and had a relatively increased usage of oral and IV medications. **(C,D)** Representative mitral, aortic and pulmonary vein Doppler velocity patterns normalized for one full cardiac cycle for each group are seen here. Patients with DCM (Clusters 3–5) have relatively abnormal Doppler tracings compared to healthy volunteers (clusters 1–2). Figure adapted from Garcia-Canadilla et al. (15).

In unsupervised machine learning, there are no labels or outcomes annotated with each observation, and instead the algorithm's task is to understand the structure of the data with tasks typically involving reducing the amount of redundant variables/features (e.g., dimensionality reduction) or quantifying the similarity between patients/observations (e.g., cluster analysis; Figure 3). Thus, the end-goal in unsupervised learning is finding relationships in the data itself. The potential of this technique to identify imaging phenotypes in echocardiography is strong due to the high dimensional and complex data generated from an echocardiogram when representing cardiac form and function (5). Unsupervised techniques are often used in combination with other techniques in a ML pipeline. For example, dimensionality reduction (e.g., principal component analysis, multiple kernel learning, autoencoders) can be used initially to identify the most important features in order to facilitate the efficiency and interpretation of a subsequent cluster analysis (15). It can also be combined with a supervised learner such as in generative adversarial networks (7). Finally, it can be used on the output of the supervised learning model to improve our understanding of how it chose to group patients (24).

**Figure 4:**
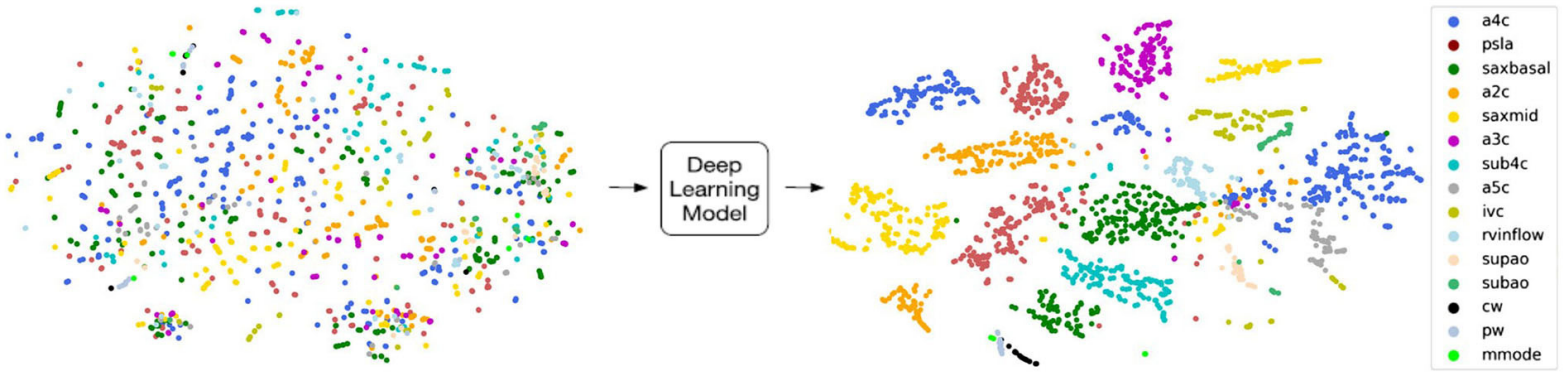
T-distributed stochastic neighbor embedding (t-sne) is a deep learning-specific dimensionality reduction visualization tool that plots how similar each image is according to the deep learning model. Madani et al. developed a convolutional neural network (CNN) to classify echocardiographic images into 18 different views, and they used t-sne to understand how the CNN analyzed each image with respect to each other. Visually distinct views tended to group farther away from each other such as continuous wave Doppler (black; cw) and apical 4 chamber view (blue; a4c) while pulse wave (gray; pw) was very similar to continuous wave and tended to overlap. Figure adapted from Madani et al. (24).

Deep learning has been very successful in performing tasks on high dimensional data and can be given both supervised and unsupervised tasks. If one considers each pixel to be an individual input feature, then an individual echocardiogram could potentially have millions of features (45). Therefore, the high dimensionality of imaging data like echocardiograms proves to be an excellent substrate for DL. The advantage of DL over traditional ML algorithms is that it can derive very complex abstract relationships for a given input with relatively less engineering of those input features. This capability is, in large part, due to several layers that analyze features in highly non-linear ways to establish mapping between the features and labels/outcomes. A major trade-off of this complexity is that it its decision-making process is increasingly abstract. Methods have been developed to specifically address explainability for this unique aspect of DL. For example, Chotzoglou et al. (12) used gradient-weighted class activation maps (Grad-CAM) to visualize which parts of an image are most important in detecting if the fetal echocardiogram was normal or abnormal (Figure 2). This provides a way for the clinician to both verify if the model decided correctly and to assist the provider in deciding if additional views are needed. As another example, Madani et al. used t-distributed stochastic neighbor embedding (t-SNE) to visualize how their deep learning model classified images into different views (Figure 4). t-SNE is a dimensionality reduction technique that has been designed to organize observations based on how similar their learned features are (i.e., activation maps) according to the DL model. Using this technique, error analysis of the mis-classified observations can facilitate understanding the strengths and inherent biases of the model. Other ways of understanding model bias include occlusion experiments and test data with artificially created image artifacts (7, 24). Finally, exploring the causal relationship between a set of features and an output is one of the goals of clinical inference (46). eXplainable AI is a field of research that attempts to reduce the issues of black-box methods including developing AI models within a framework of causality. One of its goals is to develop a “Human-AI interface” which allows the user to interrogate the model (e.g., counterfactual “what-if” questions) to gain insight into the model's decision-making process (47). While the terms interpretable and explainable are sometimes used interchangeably, some consider eXplainable AI the approach to develop models that explain why it came to its decisions while interpretable ML seeks to describe how a model came to its decision (48).

### Congenital heart disease

Since the introduction of B-mode echocardiography into clinical practice over 50 years ago (49), congenital heart specialists have honed and exceled at deriving anatomic relationships non-invasively to guide surgical decision making. Nevertheless, there are many surgical management options that rely on echocardiography where there is still clinical equipoise such as timing of neonatal Tetralogy of Fallot repair or surgical management of the patient with borderline left ventricle (LV). Most echocardiographic clinical research on borderline LV involves a reductionist approach whereby clinically accepted measures on an echocardiogram that are thought to potentially relate to prognostic information are studied to assess their strength in relating to outcomes. This approach is necessary in clinical echocardiographic research due to the abundance of information that each echo provides without a methodically robust way of identifying patterns within them. The risk scores that have been developed over the past 20 years with this approach have largely focused on aortic valve, left ventricle length, and mitral valve size (50). Yet, these heuristic-based algorithms account for some, but not all, of the complex patterns in an echocardiogram that could be useful in diagnosis (51).

Meza et al. (14) helped demonstrate that unsupervised machine learning could help identify patterns in echocardiographic data that could be clinically relevant to diagnosis and prognosis of patients with borderline left ventricle. They collected 194 functional and morphologic variables in each echocardiogram of neonates with ductal-dependent hypoplastic left-sided structures or aortic stenosis/atresia. They performed hierarchical clustering using this echo data alone without any additional clinical or outcomes data to reduce bias in understanding similarity between patients. It identified three distinct groups of patients which corresponded to multi-level LV hypoplasia, hypoplastic left heart syndrome, and critical aortic stenosis. Accordingly, surgical decision and mortality were distinguishable between groups with mortality and single ventricle palliation being the highest in the hypoplastic left heart group. Within these groups, they found that aortic valve atresia and LV end-diastolic volume best distinguished between the groups as determined by multinomial regression and linear discriminant analysis. Mitral valve characteristics and pulmonary vein anomalies, parameters often used in clinical practice to help guide clinical management, were not found to be significant in distinguishing between the three groups. This study is important in that it used echocardiographic data on congenital heart disease patients to define statistically driven variables of importance with a technique that was free of any a priori assumptions about the relationships between the variables.

### Cardiomyopathy

The manual extraction of measurements for a given set of data, like peak E wave, deceleration time, E/A ratio from a mitral inflow Doppler spectrogram, summarizes the data source into a set of expert-crafted features (e.g., Meza et al. extracted a set of 194 features). Performing manual extraction of a set of inputs can be time consuming, and subtle patterns not previously identified may be lost with this approach. To overcome this limitation, Garcia-Canadilla et al. explored the use of unsupervised learning directly on left ventricular longitudinal strain, aortic outflow Doppler, pulmonary vein Doppler, and mitral inflow Doppler velocity tracings as inputs to assess whether echocardiographic imaging phenotypes could be associated with clinical characteristics and outcomes in dilated cardiomyopathy. They used multiple kernel learning to perform dimensionality reduction and organize patients in accordance with their similarity in feature values, performed k-means cluster analysis to identify groups with similar phenotypes, and most importantly they were able to explore how each of the strain/Doppler tracings are represented in the output space. In other words, they were able to visualize how strain and Doppler velocity tracings would look like for a given group of patients. With this approach, we can potentially understand and identify how systolic and diastolic dysfunction in DCM can be represented by subtle patterns in strain and Doppler velocity data; and further, how they relate to clinical course and risk for adverse outcome. The analysis of strain and Doppler patterns rather than absolute values provides useful information. Moreover, this study used Doppler and strain data over the entire cardiac cycle, thereby providing more comprehensive data than current approaches which typically measure data at single point (e.g., peak systolic strain).

Both cluster analysis and dimensionality reduction techniques described are excellent at using underlying statistical patterns in echocardiographic data to relate observations and features to each other. However, just like in supervised learning methods, unsupervised algorithms do not necessarily make explicit how they came to their decisions. For example, cluster methods do not quantify which features are similar within a group of observations, and dimensionality reduction techniques often render feature values into abstract estimations. While the output of these unsupervised learners may be adequate for a given situation, there are methods being developed to help elucidate underlying statistical patterns including model-agnostic interpretability methods to describe feature importance in cluster analysis (52). For dimensionality reduction, separate algorithms have been developed that retain feature values after the dataset has been reduced into the low output space (CUR) (53). Finally, while the unsupervised learning techniques described in Part II are excellent at identifying key patient groups and features, they are not validated as a predictive model. Thus, in isolation, they are hypothesis generating techniques for the purposes of understanding how physiology is reflected in patterns within echocardiographic data. Unsupervised learning is often paired as a data preparation step with supervised learners and these algorithms could be used in an ML pipeline to accurately predict surgical technique (e.g., determining surgery for LV obstruction in CHD) or risk of diastolic heart failure (relating echocardiographic phenotypes of diastolic function in DCM).

## Perspectives and future steps

The non-invasive assessment of the heart through echocardiography provides us representations of the interplay between cardiac anatomy and physiology. We often perform a reductionist approach to identify the key features of an echo that are most associated with the underlying pathophysiology of the heart as it relates to clinical signs and symptoms. This approach, though easy to perform, disregards complexity and nuances in an echocardiogram that could potentially improve detection of physiologic/anatomic changes in cardiac health and disease and their association with clinical outcomes. Machine learning has been used successfully over the past few years in identifying more subtle and complex patterns in echocardiographic data that can strengthen our understanding of how cardiac (patho)physiology is represented in an echocardiogram (5). This ability to learn and categorize patterns, in conjunction with modern day computing power, provides us the ability to not only improve our understanding of cardiac anatomy and physiology, but optimize and automate logistic tasks in the clinical echocardiography laboratory.

### Challenges in pediatric cardiology

Validation of translational diagnostic tools is necessary to promote their use in clinical practice with the goal of becoming standard of care. This is especially true in pediatric cardiology where a range of anatomic variability and loading conditions, which may change over time, pose a challenge (54). Although the standard of practice in AI workflows includes a step where the model is tested on unseen data (e.g., a test or holdout set), unidentified biases and poor generalizability are still issues if the training/test set is limited in size and heterogeneity (e.g., data exclusively from developed countries, a single center, one type of imaging machine/vendor, etc.). Thus, prospective multicenter validation is still required to assess for generalizability and help identify previously unrecognized biases in the model (2, 55). Indeed, these considerations, in addition to the small sample sizes prevalent in pediatric cardiology and the inherent difficulty in translating predictive models into clinical practice, are all hurdles that need to be overcome for widespread use to occur (45, 56, 57). Ways to facilitate bias reduction and prospective validation of a model to promote widespread use includes decentralizing the AI algorithm, so-called federated learning. This developing approach is an alternative to sending de-identified data to a central storage system where the algorithm is then trained. Instead, the model is brought to each collaborating center where it is trained on the data locally which reduces the effort of data de-identification/transfer and can potentially expedite the adoption of the AI model (22). The potential downside to this approach is that quality control may be more challenging without all images being assessed and processed in a central core laboratory. Finally, while AI innovations continue to advance at a rapid pace, it is critical that the proper governance and regulatory oversight is in place to establish a secure and ethical standard (58). For example, equitable inclusion of patients during model development or maintaining high security standards against data breaches will promote acceptance of AI as a whole in the clinical community.

### AI and the clinician

Other reasons why AI is not as readily accepted in clinical practice is due to skepticism and unfamiliarity of AI with the clinician. On top of the standard rigor needed for a tool to be clinically validated, it is important that the user (i.e., the sonographer and practicing physician) is familiar with the strengths and weaknesses of the tool, is facile in its use, and can ultimately trust in its abilities. While there are conventional therapies routinely used in medicine whose mechanism of action is not fully-elucidated, understanding the process by which a diagnostic tool achieves its decision is very important in the acceptance of the model in clinical practice. Indeed, certain AI modalities (e.g., DL) are notorious for their black box nature, but the fields of interpretable machine learning and eXplainable AI have been developed to address this issue (Please see *Explainability in machine learning*). Further, it should be noted that fully automated AI have gained notoriety for not only being successful at mimicking human intelligence, but also for the critical errors that inevitably occur (e.g., the self-driving car that runs a red light). It is unlikely that fully-automated AI will exist in clinical practice without physician oversight and instead it will fulfill a much-needed role as a reliable partially automated tool (59). In addition, with the adoption of any form of automation, there is an increased risk of automation bias, an error where the user trusts the automated calculation despite overt clinical evidence suggesting it is incorrect (1). Thus, the active training of physicians to critically appraise AI models as well as training in how to use it in the clinical workspace is needed.

This review has discussed literature on optimizing and improving the clinical pediatric echocardiogram workflow. Though there is little research on this currently in pediatric echocardiography, using AI to understand clinical narratives in the echocardiogram report can both improve report consistency and enrich its diagnostic utility. Natural Language Processing (NLP) is a branch of AI and linguistics devoted to performing tasks related to speech and text, and there is a growing body of work on applying it to radiology report data (60). Tasks NLP models were designed to address include improving quality compliance by identifying if patient indications for scans adhered to study guidelines and institutional protocols (61). Many studies focused on disease surveillance including extracting relevant information for a particular disease and tracking key features longitudinally over several diagnostic reports (60).

### AI, echocardiography, and the patient

While AI has great potential to improve the standard of care in patients who are diagnosed with echocardiography, equity is an issue that needs to be accounted for early in the development of AI algorithms. Centers who perform AI research will naturally include more of their patients relative to other centers, and consequently the models will tend to be trained and tested on these populations. If not accounted for, the fitted model could make incorrect decisions if applied to a new setting due socioeconomic factors as well as variations in local practice (e.g., indications for echocardiogram, differences in image acquisition protocol) leading to significant sampling bias (22). For example, AI can improve prenatal detection of CHD especially in practices that serve disadvantaged communities where pediatric cardiology expertise may not be readily available. However, under/overrepresented medical conditions endemic to that community could impact the accuracy of the DL model if not adequately addressed (62). In addition, poor insurance coverage may limit the access to deep learning tools (62).

### AI and translational medicine

AI provides a means for rapid innovation in medicine as it is designed to perform tasks efficiently on data structures commonly used in medical research (e.g., images, video clips, and tabular datasets). However, despite all the promising results in the studies featured in this review, AI is still a translational technology which carries with it unique problems both old and new. Namely, the process of translating a novel idea into a tool used in standard of care is a complex one which involves more than just the rigorous academic stages from *in vitro* experimentation to multicenter clinical validation. Indeed, widespread clinical use of a novel technology requires regulatory approval from regional government agencies as well as industry partnership to help facilitate the accessibility of the technical innovation. It is impossible for one person to be expert in all of these facets of translational medicine, and thus a multidisciplinary team of collaborators who partner well between clinicians, academia, industry, and government is needed to shepherd these novel AI tools to clinical implementation (63).

## Conclusion

The echocardiogram is the first-line imaging tool for the cardiologist due to its ability to allow the clinician to quickly identify cardiac anatomy and physiology. With the advent of AI in medical imaging, we can extend the utility of a cardiac ultrasound beyond what is immediately apparent to explore patterns previously unseen and make diagnoses more accurately and efficiently. Indeed, based on current trends, we expect that the next era of pediatric echocardiography will be data-centric where AI will augment and integrate the role of the sonographer, echocardiographer, and clinician to improve patient care. The development of the AI-based pediatric echocardiography laboratory of the future will however be a long path with many expected obstacles, given the complexity of pediatric heart disease.

## Author contributions

MN and LM contributed to the design of the study. MN and OV wrote the first draft of the manuscript. MF, LL, CR, and LM wrote sections of the manuscript. MN and LM performed final revisions of the manuscript. All authors contributed to the article and approved the submitted version.

## Conflict of interest

The authors declare that the research was conducted in the absence of any commercial or financial relationships that could be construed as a potential conflict of interest.

## Publisher's note

All claims expressed in this article are solely those of the authors and do not necessarily represent those of their affiliated organizations, or those of the publisher, the editors and the reviewers. Any product that may be evaluated in this article, or claim that may be made by its manufacturer, is not guaranteed or endorsed by the publisher.
